# Randomised clinical trial: intravenous vs oral iron for the treatment of anaemia after acute gastrointestinal bleeding

**DOI:** 10.1111/apt.15327

**Published:** 2019-06-14

**Authors:** Luis Ferrer‐Barceló, Laura Sanchis Artero, Javier Sempere García‐Argüelles, Pilar Canelles Gamir, Javier P. Gisbert, Luis Manuel Ferrer‐Arranz, Ana Monzó Gallego, Lydia Plana Campos, Jose Mª Huguet Malavés, Marisol Luján Sanchis, Lucía Ruiz Sánchez, Susana Barceló Cerdá, Enrique Medina Chuliá

**Affiliations:** ^1^ Hospital General Universitario de Valencia Servicio de Patología Digestiva Valencia Spain; ^2^ Hospital Universitario de La Princesa, Instituto de Investigación Sanitaria Princesa (IIS‐IP) y Centro de Investigación Biomédica en Red de Enfermedades Hepáticas y Digestivas (CIBERehd) Madrid Spain; ^3^ Departamento de Estadística e Investigación Operativa y Calidad Universitat Politècnica de València Valencia Spain

## Abstract

**Background:**

Acute gastrointestinal bleeding is prevalent condition and iron deficiency anaemia is a common comorbidity, yet anaemia treatment guidelines for affected patients are lacking.

**Aim:**

To compare efficacy and safety of intravenous ferric carboxymaltose (FCM) and oral ferrous sulphate (FeSulf) in patients with anaemia secondary to non‐variceal gastrointestinal bleeding

**Methods:**

A prospective 42‐day study randomised 61 patients with haemoglobin <10 g/dL upon discharge (Day 0) to receive FCM (n = 29; Day 0: 1000 mg, Day 7: 500 or 1000 mg; per label) or FeSulf (n = 32; 325 mg/12 hours for 6 weeks). Outcome measures were assessed on Days 0 (baseline), 7, 21 and 42. The primary outcome was complete response (haemoglobin ≥12 g/dL [women], ≥13 g/dL [men]) after 6 weeks.

**Results:**

A higher proportion of complete response was observed in the FCM vs the FeSulf group at Days 21 (85.7% vs 45.2%; *P* = 0.001) and 42 (100% vs 61.3%; *P* < 0.001). Additionally, the percentage of patients with partial response (haemoglobin increment ≥2 g/dL from baseline) was significantly higher in the FCM vs the FeSulf group (Day 21:100% vs 67.7%; *P* = 0.001, Day 42:100% vs 74.2%; *P* = 0.003). At Day 42, normalisation of transferrin saturation to 25% or greater was observed in 76.9% of FCM vs 24.1% of FeSulf‐treated patients (*P* < 0.001). No patient in the FCM group reported any adverse event vs 10 patients in the FeSulf group.

**Conclusion:**

FCM provided greater and faster Hb increase and iron repletion, and was better tolerated than FeSulf in patients with iron deficiency anaemia secondary to non‐variceal acute gastrointestinal bleeding.

## 1 INTRODUCTION

Acute gastrointestinal bleeding (GIB) is a prevalent condition (90‐108 cases per 100 000 incidence) with a substantial economic burden and is a direct cause of substantial mortality (3%‐14%).^1^, ^2^

Iron deficiency anaemia (IDA) following non‐variceal upper GIB is estimated to occur in approximately 50% of all cases. Notably, mortality among patients with upper GIB is mostly related to the decompensation of underlying diseases, in which anaemia can play a key role.^3^ The treatment of GIB‐associated anaemia, therefore, requires both correction of the underlying cause and adequate repletion of iron stores in case of IDA.^4^ Despite the known frequency of GIB and the negative consequences of IDA, guidelines for the effective treatment of GIB‐associated anaemia remain poorly defined.^5^

Oral administration of ferrous sulphate (FeSulf) is still re‐cognised as the first‐line treatment for IDA patients. However, for patients with gastrointestinal disorders, oral iron shows substantial limitations due to insufficient absorption, slow course of action and severe gastrointestinal side effects that can exacerbate existing symptoms.^5^, ^6^, ^7^ Due to this intolerance, it is estimated that oral iron treatment is discontinued in up to 50% of IDA patients.^8^

For patients with poor tolerance of oral iron, a large iron deficit or need for rapid response to treatment, intravenous (iv) iron admi‐nistration is the treatment of choice.^9^, ^10^, ^11^ Intravenous iron is admi‐nistered as an iron‐carbohydrate complex, and various formulations that can be given at different maximum doses or minimum administration times (mainly depending on the product's stability and premature release of iron) are currently available.^12^, ^13^ By bypassing the gut, iv iron circumvents slow absorption associated with oral iron, promotes rapid uptake by the reticuloendothelial system and prevents gastrointestinal inflammation.^14^ Additionally, large doses of iv iron can be administered over a short period of time, facilitating a rapid and long‐lasting response. Overall, iv iron administration is associated with improved treatment compliance and reduced demand for red blood cell transfusions, which is highly recommended given their scarcity, cost and potential risks.^2^, ^6^, ^15^, ^16^, ^17^

The use of iv iron has been well studied for the treatment of IDA associated with a wide range of underlying digestive pathologies, including inflammatory bowel disease, coeliac disease and colorectal cancer as well as non‐digestive haemorrhagic events.^8^, ^18^, ^19^, ^20^, ^21^, ^22^, ^23^, ^24^ However, only few studies have analysed the efficacy of different iron regimens in patients with IDA secondary to acute GIB and even less for patients with lower GIB. One randomised study in patients with upper GIB compared a fixed single dose of iv iron (ferric carboxymaltose, FCM) vs oral iron (FeSulf). While the increase of haemoglobin (Hb) was independent of the iron administration route, the correction of iron deposits was more rapidly achieved by iv FCM than oral FeSulf.^25^ A recently published retrospective analysis of patients who were admitted with acute GIB and treated with FCM either alone or combined with red blood cell transfusions suggested effectiveness and safety of FCM in this population.^26^

The randomised study presented here compared the efficacy and safety of iv FCM given at weight‐ and Hb‐adjusted doses according to the summary of product characteristics (SmPC) and oral FeSulf in patients with anaemia secondary to non‐variceal acute GIB, aiming to improve the evidence base in lieu of established treatment protocols.

## 2 MATERIALS AND METHODS

### 2.1 Patients

Clinically stable patients admitted to the University General Hospital of Valencia, Spain, with non‐variceal GIB (aged >18 years) and subsequent diagnosis of anaemia secondary to acute GIB (Hb <10 g/dL on the day of hospital discharge, Day 0) who did not meet the exclusion criteria were enrolled into the trial. GIB was defined as any sign or symptom of macroscopic exteriorisation of haemorrhage accompanied by changes in relevant laboratory values (eg decrease of Hb). Obscure origin GIB was defined as exteriorisation of macroscopic anal haemorrhage of undefined cause after gastroscopy and colonoscopy.

Exclusion criteria were pathologies that could influence anaemia progression (chronic renal failure, liver disease, inflammatory bowel disease, neoplasia, uncompensated thyroid disorders, malabsorption, HIV, haematological disorders, folic acid or vitamin B12 deficiency and previous gastric surgery). Further exclusion criteria were non‐iron deficiency anaemia or anaemia of mixed origin, a history of chronic anaemia, treatment with erythropoietin, iron supplements, folic acid or vitamin B12 in the year prior to inclusion and contraindications to treatment with study drugs. In addition, available clinical history and laboratory data of patients covering one year prior to inclusion were reviewed to exclude patients with undiagnosed iron deficiency prior to the GIB that lead to hospital admission.

The criteria for withdrawal from the study were relapse of GIB or requiring transfusion after hospital discharge.

### 2.2 Study design

The study was designed as a single centre, prospective, unblinded study and was conducted in accordance with the Helsinki Declaration and adherence to Good Clinical Practice guidelines. The study was approved by the Spanish Health Authorities and the ethics committee of the University General Hospital of Valencia, and was registered in the European Clinical Trials Database (EudraCT number 2016‐002660‐13). A signed informed consent was required for inclusion.

On the day of hospital discharge (Day 0), eligible patients were randomised 1:1 (alternating sequence in order of enrolment controlled by the principle investigator) to treatment with iv FCM or oral FeSulf. Treating and monitoring physicians were not aware which treatment patients would receive after discharge. Outcome measures were assessed on Day 0 (baseline), 7, 21 and 42 of the study. Adverse events and treatment adherence were monitored throughout the study.

Blood samples were taken at each visit to determine Hb, transferrin saturation (TSAT), ferritin, alanine transaminase and aspartate transaminase blood levels. Surveys on patients’ quality of life used EuroQoL 5 Dimensions (EQ‐5D‐3L) questionnaires in Spanish,^27^ and were conducted face to face or via telephone on Day 0, 7, 21 and 42. At each visit, patients were asked for treatment adherence. Patients declaring that they took >90% of tablets were considered adherent. No patient diaries were used, but patients were asked how many tablets they had left in the box. In case of non‐adherence, patients were asked if it was due to adverse effects.

### 2.3 Treatment characteristics

Patients in the iv FCM group received iron dosages of 1500 or 2000 mg (Ferinject^®^, Vifor France SA, Paris, France) dependent on body weight according to the SmPC, with a first dose of 1000 mg iron on Day 0 and a second dose of either 500 or 1000 mg on Day 7.

Patients randomised to oral FeSulf (Fero‐Gradumet^®^, Teofarma SRL, Pavia, Italy) received an iron dosage of 650 mg/d (325 mg BID for 6 weeks, commencing on Day 0).

No vitamins or dietary supplements were given throughout the study duration.

Patients who did not show Hb normalisation at the end of the study were treated according to local clinical practice (ie iv FCM for patients not responding to oral FeSulf).

### 2.4 Outcome measures

The primary outcome measure was complete response rate (percent of patients who reached Hb levels ≥12 g/dL or ≥13 g/dL in women and men respectively) at Day 42. Secondary outcome measures included partial response rate (percentage of patients with Hb increase ≥2 g/dL vs baseline at Day 7, 21 or 42), the rate of iron status normalisation (percentage of patients who achieved TSAT ≥25%; centre‐specific cut‐off for normal TSAT) and serum ferritin levels.

Adverse events were considered treatment related when they were described in the drugs’ SmPCs and chronologically related to the treatment administration (ie the first 24 hours after infusion for FCM and the entire 42‐day treatment period for oral iron).

### 2.5 Statistics

The sample size was calculated on the premise that an increase in Hb of 0.5 g/dL per 100 mg of absorbed iron and a minimum Hb increase of 1.6 g/dL would be clinically relevant. Comparison of both groups utilised a standard deviation of 2, an alpha value of 0.05 and expected power of 80%. Thus, with an estimated 15% loss to follow‐up, each group needed to include 29 patients completing the study.

For the analysis of the categorical variables (complete response and partial response), the response rates were calculated and Fisher exact test or chi‐square test was used. For the comparison of the quantitative parametric variables (demographic and analytical des‐criptive variables), mean values and standard deviation (SD) were calculated followed by application of Student's *t* test. In case of quantitative non‐parametric variables, the Mann‐Whitney *U* test (Wilcoxon Rank Sum Test) was applied.

## 3 RESULTS

### 3.1 Patient characteristics

**Figure 1:**
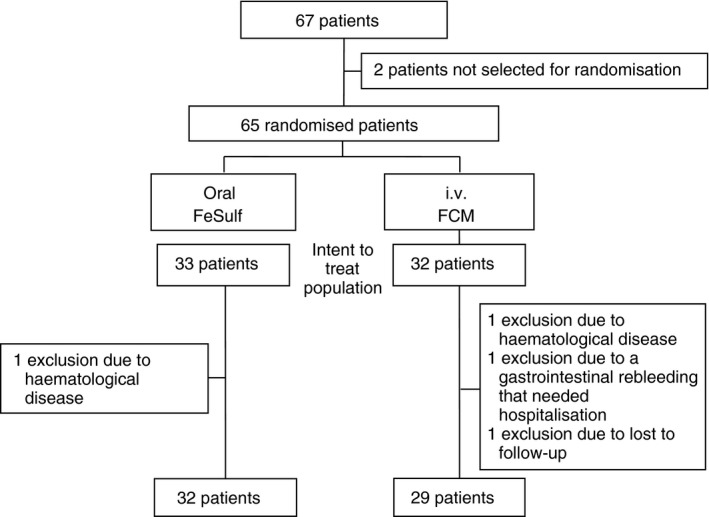
Patient flow CONSORT diagram. FCM, ferric carboxymaltose; FeSulf, ferrous sulphate

Of 67 patients hospitalised with acute GIB, 65 were randomised (32 to iv FCM, 33 to oral FeSulf; Figure 1). Of those, four patients (three in the FCM group, one in the FeSulf group) who have received at least one dose of the study drug were excluded from the intent‐to‐treat analysis. Overall, the aetiology of GIB was peptic ulcer (n = 33; 54%), obscure origin GIB (n = 7; 11.5%) lower GIB due to diverticula (n = 6; 10%), Dieulafoy's lesion (n = 4; 7%), Mallory Weiss syndrome (n = 3; 5%), lower GIB post polypectomy (n = 3; 5%) or other causes (n = 5; 8.2%).

Baseline characteristics and laboratory measures were comparable between groups (Table 1). Mean (SD) Hb levels and TSAT in the FeSulf and FCM group at baseline (hospital discharge) were 9.2 (±0.7) vs 9.3 (±0.5) g/dL and 14.9% (±8.9) vs 16% (±12.5) respectively (differences were not statistically significant).

**Table 1:** Demographic data and baseline^a^ clinical characteristics

	Oral FeSulf (N = 32)	iv FCM (N = 29)	*P*
Age (mean, y ± SD)	62.5 ± 18.3	57.8 ± 15.3	0.284
Sex (n [%])			
Men	22 (68.7%)	17 (58.6%)	0.411
Women	10 (31.3%)	12 (41.4%)	
Body weight (mean, kg ± SD)	76.9 ± 16.4	72.5 ± 10.5	0.261
Underlying causes of GIB (n [%])			0.22^b^
Duodenal ulcer	7 (21.9%)	11 (37.9%)	
Gastric ulcer	9 (28.1%)	5 (17.2%)	
Obscure origin GIB	3 (9.4%)	4 (13.8%)	
Diverticular lower GIB	5 (15.6%)	1 (3.4%)	
Dieulafoy's lesion	4 (12.5%)	0 (0.0%)	
Post‐polypectomy lower GIB	1 (3.1%)	2 (6.9%)	
Mallory Weiss	2 (6.3%)	1 (3.4%)	
NSAID‐related lower GIB	0 (0.0%)	1 (3.4%)	
Gastric and duodenal ulcer	0 (0.0%)	1 (3.4%)	
Ulcerated submucosal benign tumour	0 (0.0%)	1 (3.4%)	
CMV rectal ulcer	0 (0.0%)	1 (3.4%)	
Antral vascular ectasia	1 (3.1%)	0 (0.0%)	
Erosive gastritis of hernia	0 (0.0%)	1 (3.4%)	
Co‐medications (%)			
Anticoagulants	6 (18.8)	6 (20.7)	0.849
Antiplatelets	5 (15.6)	9 (31.0)	0.153
Proton pump inhibitors	9 (28.1)	13 (44.8)	0.175
Hb at hospital admission (mean, g/dL ± SD)	9.7 ± 2.6	9.4 ± 2.6	0.686
Red blood cell units at hospital admission (median, Q1‐Q3)	1.5 (0‐4)	2 (0‐3)	0.843
Transfused patients (%)	56.3	55.2	0.933
Length of stay from hospital admission to Day 0 (mean, days ± SD)	5.26 ± 2.73	5.66 ± 3.18	0.605
Hb at hospital discharge^c^ (mean, g/dL ± SD)	9.2 ± 0.7	9.3 ± 0.5	0.617
TSAT at hospital discharge^c^(mean, % ± SD)	14.9 ± 8.9	16 ± 12.5	0.678
Ferritin at hospital discharge^c^(mean, μg/L ± SD)	78.5 ± 62.2	85.4 ± 82.2	0.712
Patients with iron deficiency^d^ (n [%])	29 (90.6)	23 (79.3)	0.454

Abbreviations: CMV, cytomegalovirus; FCM, ferric carboxymaltose; FeSulf, ferrous sulphate; GIB, gastrointestinal bleeding; Hb, haemoglobin; NSAID, non‐steroidal anti‐inflammatory drug; Q, quartile; SD, standard deviation; TSAT, transferrin saturation.

a Baseline data were taken on the day of hospital discharge.

b No statistically significant differences in the causes of GIB between groups

c Laboratory data before treatment drug administration (Day 0).

d Iron deficiency defined as TSAT <25%.

### 3.2 Efficacy outcomes

#### 3.2.1 Hb response

**Figure 2:**
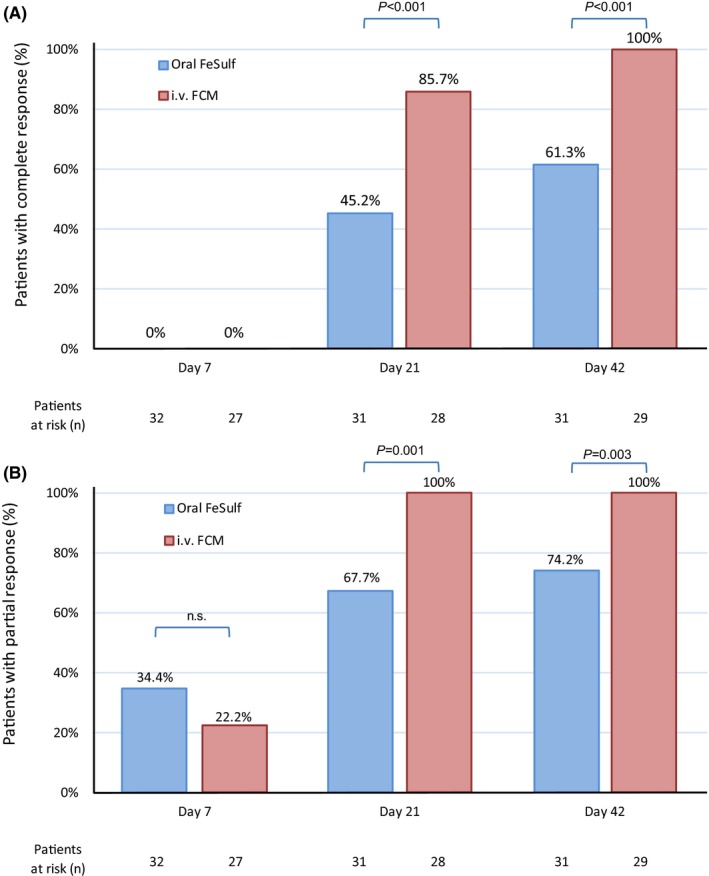
Complete (A) and partial (B) response of patients after iv FCM or oral FeSulf administration over the duration of the study. FCM, ferric carboxymaltose; FeSulf, ferrous sulphate

**Figure 3:**
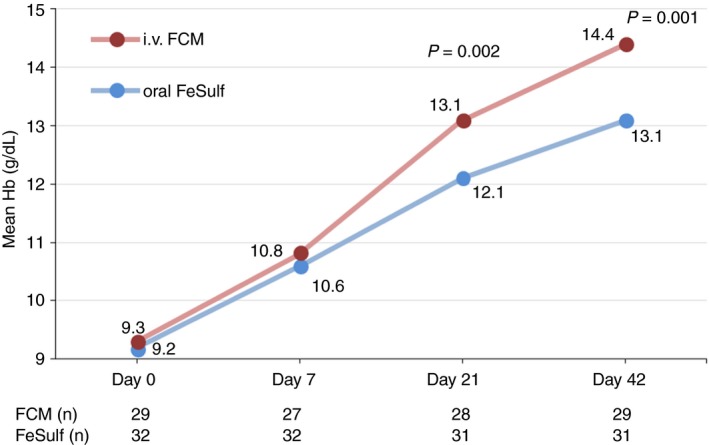
Mean Hb levels of patients treated with iv FCM or oral FeSulf. Lab measurements on Day 0 were done before treatments were administered. FCM, ferric carboxymaltose; FeSulf, ferrous sulphate; Hb, haemoglobin

At the end of the study (Day 42), complete response was achieved by 100% of FCM‐treated patients compared with 61.3% of oral FeSulf‐treated patients (*P* < 0.001) (Table 2 and Figure 2). At Day 21, the percentage of complete responders was almost twice in the FCM group that compared with the FeSulf group (85.7% vs 45.2%; *P* = 0.001). Similarly, a significantly greater proportion of FCM‐ than FeSulf‐treated patients achieved a partial response at Day 21 and 42 (100% vs 67.7%; *P* = 0.001 and 100% vs 74.2%; *P* = 0.003, respectively). The numerical difference in partial response rate on Day 7 was not statistically significant. The time course of Hb values throughout the study period is illustrated in Figure 3.

**Table 2:** Comparison of outcome measures

	Day 7			Day 21			Day 42		
	Oral FeSulf	iv FCM	*P*	Oral FeSulf	iv FCM	*P*	Oral FeSulf	iv FCM	*P*
Complete response^a^	0% (0/32)	0% (0/27)	—	45.2% (14/31)	85.7% (24/28)	0.001	61.3% (19/31)	100% (29/29)	0.001
Partial response^b^	34.4% (11/32)	22.2% (6/27)	0.231	67.7% (21/31)	100% (28/28)	0.001	74.2% (23/31)	100% (29/29)	0.003
% with iron repletion^c^	19.4% (6/31)	44.4% (12/27)	0.039	22.6% (7/31)	46.2% (12/26)	0.055	24.1% (7/29)	76.9% (20/26)	<0.001
TSAT (mean, % ± SD)	17 ± 12.6 (n = 31)	25.7 ± 10.7 (n = 27)	0.007	17.3 ± 9.8 (n = 31)	27.1 ± 8.0 (n = 26)	<0.001	19.3 ± 8.8 (n = 29)	30.3 ± 6.9 (n = 26)	<0.001
Ferritin (mean, μg/L ± SD)	67 ± 47 (n = 32)	673 ± 184 (n = 27)	<0.001	55 ± 39 (n = 31)	596 ± 267 (n = 26)	<0.001	62 ± 50 (n = 28)	384 ± 211 (n = 26)	<0.001

Note

Response rates and rates of patients with iron repletion shown as percentages and number of patients with achievement per all patients with relevant data.

Abbreviations: FCM, ferric carboxymaltose; FeSulf, ferrous sulphate; SD, standard deviation; TSAT, transferrin saturation.

a Complete response: Hb ≥12 and 13 g/dL in women and men, respectively.

b Partial response: Hb increment ≥2g/dL from baseline.

c Iron repletion defined as TSAT ≥25%.

#### 3.2.2 Iron availability

**Figure 4:**
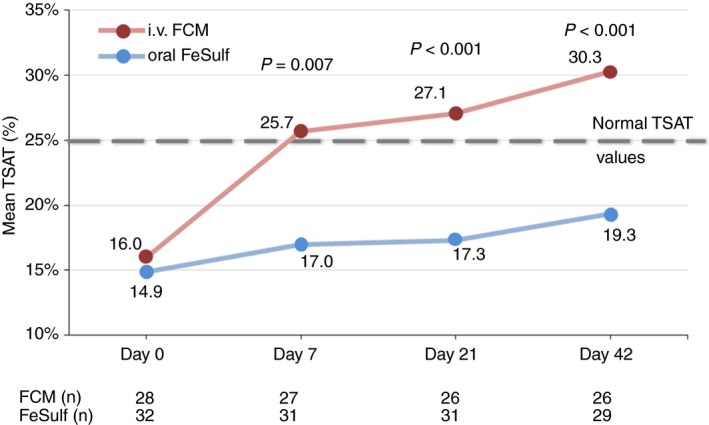
Mean transferrin saturation index of patients treated with iv FCM or oral FeSulf. Lab measurements on Day 0 were done before treatments were administered. FCM, ferric carboxymaltose; FeSulf, ferrous sulphate; TSAT, transferrin saturation

Mean TSAT values were significantly greater in FCM‐ than FeSulf‐treated patients on Day 7, 21 and 42 (*P* = 0.007, *P* < 0.001 and *P* < 0.001, respectively; Figure 4). Mean TSAT was >25% in the FCM group but <25% in the FeSulf group at all post‐baseline visits. At Day 42, normal TSAT (>25%) was achieved by 76.9% of FCM‐treated vs 24.1% of FeSulf‐treated patients (*P* < 0.001, Table 2).

Mean serum ferritin levels increased rapidly in the FCM group and remained >100 µg/L from Day 7‐42 (Table 2).

### 3.3 Quality of life

**Figure 5:**
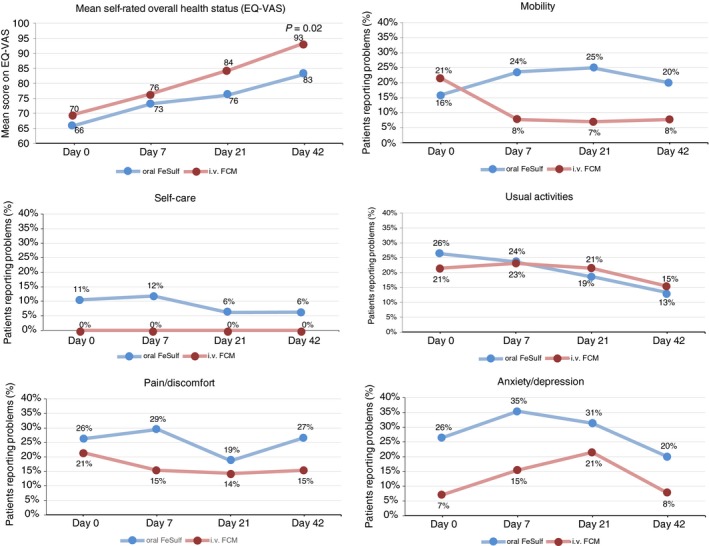
Results of quality of life questionnaire EQ‐5D‐3L and EQ‐VAS. For EQ‐5D‐3L dimensions (mobility, self‐care, usual activities, anxiety/depression and pain/discomfort) higher percentages correspond to more patients with problems. For EQ‐VAS, higher scores correspond to better health status (quality of life). FCM, ferric carboxymaltose; FeSulf, ferrous sulphate

EQ‐5D‐3L quality of life questionnaires were completed by 33 patients (14 from the FCM group, 19 from the FeSulf group). Serial analysis of quality of life questionnaires at Day 0, 7, 21 and 42 showed a numerical decrease in the percentage of patients with mobility problems and with pain‐discomfort in the FCM group compared with an increase in the FeSulf group (Figure 5). The other dimensions of EQ‐5D‐3L showed similar time courses for the two groups. At the end of the study (Day 42), the subjective quantitative measure of overall health status (EQ‐VAS scale) was significantly better in FCM‐treated compared with FeSulf‐treated patients (*P* = 0.02).

### 3.4 Tolerability

No treatment‐related adverse events, withdrawals or dose reductions were reported for the FCM group. Headache and elevated alanine transaminase levels (less than 1.5 times of normal values) were reported in one (3.1%) patient each (on Day 7 and 21) and constipation in two (6.2%) patients (two on Day 21 and one who remains on Day 42).

In FeSulf‐treated patients, adverse events were reported for 10 (30.3%) patients, all of them considered treatment‐related adverse events. Constipation was reported for 7 (21.2%) patients on Day 7 and 21 and an additional patient on Day 42. Two patients (6.1%) reported abdominal pain on Day 21 (one of those resulting in dose reduction by half) but only one (3%) on Day 42.

No side effects were reported during the first administration of the study drug (Day 0).

### 3.5 Adherence to treatment

All FCM‐treated patients adhered to treatment compared with 84.4% in the FeSulf group (*P* = 0.03). Non‐adherence to oral FeSulf was due to abdominal pain and related dose reduction or forgetting to take the treatment. Notably, two patients in the FeSulf group required FCM rescue medication due to very low Hb levels (8 and 8.9 g/dL) on Day 21.

## 4 DISCUSSION

Our study shows that patients with anaemia after acute non‐variceal GIB respond significantly better to iv FCM given at an Hb‐ and body weight‐depending dose scheme (as per SmPC) compared with high‐dose oral FeSulf. Treatment with FCM resulted in rapid normalisation of mean Hb levels at 3 weeks after hospital discharge, with normalised Hb levels in 85% of FCM‐ compared with 45% of FeSulf‐treated patients and 100% vs 60% with complete response at the end of the study. Partial response was reached in a 100% of patients with FCM at 3 weeks and 6 weeks, the end of the study, compared with 67% and 74%, respectively, with oral FeSulf. Furthermore, FCM was superior to FeSulf in normalising TSAT (utilisable iron) and serum ferritin (iron stores) from Day 7 throughout the entire 42‐day study period.

In 2014, Bager et al showed the efficacy of oral and iv iron supplementation for the treatment of anaemia secondary to upper GIB in a placebo‐controlled trial. When comparing the outcomes between the two iron supplementation groups, improvements in Hb did not differ significantly whereas the correction of iron deposits was more rapidly achieved by iv FCM than oral iron.^25^ One reason for the different outcomes in correcting Hb levels and iron stores may be that FCM was administered as a single dose of 1000 mg iron, which can be insufficient to cover the iron requirement in some patients. Furthermore, patients in the study by Bager et al had less severe anaemia and IDA than the patients in the study presented here (mean Hb at baseline 10.1 and 9.7 g/dL in the oral and iv iron group respectively; mean ferritin 174 and 161 μg/L; mean TSAT 21% and 20%). Studies comparing iv FCM and oral FeSulf in other indications with acute and chronic blood loss (eg uterine bleeding, postpartum anaemia) using a similar study design as the study presented here showed superiority of FCM in the correction of both anaemia and iron deficiency.^10^, ^28^, ^29^, ^30^

A recently published retrospective analysis reported data from patients with acute GIB who were treated with a single 1000 mg iron dose FCM either alone or combined with red blood cell transfusions (given in patients with Hb <7 g/dL or haemodynamic instability).^26^ Patients in both the FCM + transfusion and the FCM alone group recovered from lowest in‐hospital Hb levels of 7.2 and 8.8 g/dL to 9.4 and 9.3 g/dL at discharge and to 12.4 and 13.7 g/dL at 2‐month follow‐up, suggesting that FCM therapy can facilitate a restrictive transfusion policy. Notably, improvements in subgroups of elderly (≥75 years) and patients with more comorbidities (Charlson Index ≥ 3) were similar as in the overall study population.

The results of our study showing the superiority of FCM over oral FeSulf for the treatment and resolution of anaemia secondary to acute GIB is in line with evidence for the efficacy and safety of FCM in a wide range of conditions such as chronic kidney disease, colorectal cancer, inflammatory bowel disease, uterine bleeding and postpartum anaemia.^10^, ^18^, ^20^, ^21^, ^24^, ^29^, ^31^, ^32^, ^33^, ^34^, ^35^, ^36^ The results are also consistent with a meta‐analysis that assessed studies using different iv iron preparation to treat IDA in patients with different conditions and concluded that although all iv and oral formulations could correct IDA, FCM was the superior treatment.^37^

The rapid increase in serum ferritin as well as TSAT is typical for the rapid uptake and utilisation of intravenously administered iron, bypassing the slow and limited enteral absorption of oral iron. As it is known that iv iron rapidly increases ferritin, this is the reason for high observed ferritin levels and not an inflammatory background as basal values were normal. Furthermore, our study assessed ferritin levels early, which therefore cannot be compared with the assessment at 13 weeks by Bager et al. Notably, mean serum ferritin levels in our study remained above 100 µg/L among FCM‐treated patients until the end of the study. Conversely, mean ferritin levels remained unchanged and below 100 µg/L in the FeSulf group during the entire study period.

Prior to initiation of study treatment, that is, the period between hospital admission and discharge (Day 0), 55.7% of patients received red blood cell transfusions, yet mean Hb levels did not improve but slightly decreased. Transfusions were considered when Hb levels decreased below 7.5‐8.0 g/dL, depending on the clinical needs of the patients. This Hb cut‐off may seem high compared with restrictive transfusion protocols that emerged during the conduct of the study.^38^ However, such restrictive cut‐offs are proposed for patients without risk factors, whereas higher Hb cut‐offs may be considered for patients at risk of organ dysfunction or haemodynamic instability.^39^ In any case, decisions about the use of transfusion during this period were not influenced by the study because inclusion into the study was decided on the day of hospital discharge.

In our study, treatment with FCM was well tolerated during administration and the reported events were without clinical reper‐cussion. Based on a terminal half‐life of 7‐12 hours for the clearance of FCM and its average residence time of 11‐18 hours,^40^ observations of potential drug‐related events were only expected in this early period during/after iv administration (Day 0‐7). No acute effects were reported in this period and despite frequent assessment of potential adverse events on Days 0, 7, 21 and 42 over the 42‐day study period only minor events that were considered unrelated to FCM were observed. Even sensitive assessment of liver function (by the measurement of aspartate transaminase and alanine transaminase) showed only two recorded elevations of alanine transaminase that were below 1.5 times the upper limit of normal and considered not clinically relevant. Conversely, in the FeSulf group, a substantial proportion of gastrointestinal adverse events was detected in the FeSulf group, which even forced the reduction of the FeSulf dose by half in one of the patients. The association of gastrointestinal adverse events with oral iron administration is known from several studies.^19^, ^21^, ^41^, ^42^, ^43^, ^44^

Consistent with the good tolerability of FCM, adherence to treatment was 100%. The adherence observed in the FeSulf group (84%) was lower compared to the FCM group but still high compared to prior published studies.^8^, ^45^ However, this high percentage is based on patients’ self‐declaration when they were asked for adherence during examinations, and therefore, non‐adherence may be underreported.

Subjective state of health at the end of the study as assessed by EQ‐VAS scale was significantly better in FCM‐treated compared with FeSulf‐treated patients. To our knowledge, this is the only currently available study reporting quality of life in patients with GIB treated for iron deficiency anaemia. However, the results are consistent with other comparative trials in patients with anaemia secondary to gynaecological conditions or inflammatory bowel disease, showing a correlation between correction of anaemia and improvement of quality of life.^46^, ^47^ Although no great differences in the dimensions self‐care and usual activities of patients were observed between both treatment groups, there seems to be a higher percentage of patients in the oral FeSulf group who reported problems in mobility, pain‐discomfort and anxiety dimensions. FCM‐treated patients showed better outcome in subjective state of overall health status.

Although the primary and most secondary outcome measures were based on objective laboratory parameters, the unblinded design of this study and the ‘quasi random’ alternative sequence of enrolment may have influenced patient care, classification of adverse events and the conduct and interpretation of the quality of life surveys. The risk of selection bias was minimised by keeping physicians who investigated patients prior a potential hospital discharge (day of randomisation) unaware about the consecutive study treatment. An explorative, unadjusted post hoc subgroup analysis revealed no statistically significant difference in the rate of Hb‐normalisation at Day 42 between patients with or without transfusions during hospitalisation (73.5% vs 88.5%; *P* = 0.201).

While cost‐effectiveness was not an endpoint of this study, a recently published cohort study of patients with gastrointestinal disease and IDA suggested a potential advantage of iv over oral iron in terms of healthcare utilisation due to lower risk of hospital re‐attendance and shorter length of stay.^48^ Our study showing rapid correction of both anaemia and iron deficiency among FCM‐treated patients with Hb levels <10 g/dL can support the identification of a feasible cut‐off when to prefer iv over oral iron in patients with gastrointestinal disease.

In conclusion, this study demonstrates that in patients with anaemia after acute gastrointestinal bleeding, iv ferric carboxymaltose offers a faster and more efficient normalisation of haemoglobin and iron status parameters compared to the oral administration of ferrous sulphate. Additionally, the administration of ferric carboxymaltose displays a low profile of adverse events and improved patient quality of life, further contributing to an overall favourable benefit‐risk profile. Overall, the results of this study could provide clinical support for the inclusion of ferric carboxymaltose into treatment schemes and guidelines for patients with anaemia secondary to acute haemorrhage of gastrointestinal origin.

## AUTHORSHIP

*Guarantor of the article*: Luis Ferrer‐Barceló, MD, takes responsibility for the integrity of the work as a whole, from inception to published article.

*Author contributions*: Luis Ferrer‐Barceló, Pilar Canelles Gamir, Javier P. Gisbert and Susana Barceló Cerdá designed the study; Luis Ferrer‐Barceló, Laura Sanchis Artero, Javier Sempere García‐Argüelles, Pilar Canelles Gámir, Luis Manuel Ferrer Arranz, Ana Monzó Gallego, Lydia Plana Campos, Jose Mª Huguet Malavés, Marisol Luján Sanchis, Lucía Ruiz Sánchez and Enrique Medina Chuliá were involved in research and collection of data. Luis Ferrer‐Barceló, Pilar Canelles Gamir, Javier P. Gisbert and Susana Barceló Cerdá were involved in the analysis and interpretation of data; Luis Ferrer‐Barceló, Pilar Canelles Gamir and Javier P. Gisbert wrote and revised the manuscript. All authors reviewed and approved the final version of the manuscript.
